# Estrogenic Evaluation and Organochlorine Identification in Blubber of North Sea Harbour Porpoise (*Phocoena phocoena*) Stranded on the North Sea Coast

**DOI:** 10.1155/2015/438295

**Published:** 2015-05-17

**Authors:** Pedro Henrique Imazaki, François Brose, Thierry Jauniaux, Krishna Das, Marc Muller, Marie-Louise Scippo

**Affiliations:** ^1^Laboratory of Food Analysis, Department of Food Science, FARAH-Veterinary Public Health, University of Liège, Bâtiment B43b, Quartier Vallée 2, Avenue de Cureghem 10, 4000 Liège, Belgium; ^2^Department of Morphology and Pathology, FARAH-Veterinary Public Health, University of Liège, Bâtiment B43, Quartier Vallée 2, Avenue de Cureghem 6, 4000 Liège, Belgium; ^3^Laboratory for Oceanology, MARE Center, University of Liège, Bâtiment B6c, Quartier Agora, Allée du Six Août 11, 4000 Liège, Belgium; ^4^Organogenesis and Regeneration, GIGA-R, University of Liège, Bâtiment B34, Quartier Hôpital, Avenue de l'Hôpital 1, 4000 Liège, Belgium

## Abstract

Thirteen individual organochlorine compounds at 3 concentrations (80, 400, and 2000 ng/mL culture medium), as well as mixtures, were assayed for the estrogen receptor (ER) activation or inhibition, using a luciferase reporter gene assay (RGA). None of the PCB 138, 153, or 180 or their mixture induced a response in the RGA.* o*,*p*′-DDT was the most potent xenoestrogen from the DDT group, inducing a response already at 80 ng/mL. From the HCH and HCB group, only *β*-HCH (at 400 and 2000 ng/mL) and *δ*-HCH (at 2000 ng/mL) displayed estrogenic activities. These 13 organochlorines were determined by GC-MS in 12 samples of North Sea harbor porpoise blubber. The PCBs were the main contaminants. Within each group, PCB 153 (6.0 × 10^2^~4.2 × 10^4^ *μ*g/kg),* p*,*p*′-DDE (5.1 × 10^2^~8.6 × 10^3^ *μ*g/kg), and HCB (7.6 × 10^1^~1.5 × 10^3^ *μ*g/kg) were the compounds found in highest concentrations. The hormonal activity of the porpoise blubber samples was also assayed in RGA, where two samples showed estrogenic activity, seven samples showed antiestrogenic activity, and one sample showed both estrogenic and antiestrogenic activity. Our results suggest that the 13 POPs measured by GC-MS in the samples cannot explain alone the estrogenicity of the extracts.

## 1. Introduction

The harbour porpoise (*Phocoena phocoena*) is the most common cetacean species in the North Sea [1], and there is a growing concern about the adverse effects of persistent environmental contaminants on this and other marine mammal species [2]. Since 1998, the southern region of the North Sea has been characterised by an increased number of stranded marine mammals, in particular the harbour porpoise. However, a temporary increase in the porpoise population in the southern North Sea may have been responsible [3, 4]. Since marine mammals are at the top of the aquatic food chain and have rather long lifespans, they are an important tool to check the long-term effects concerning marine environment pollution and can be studied as global pollution indicators as well [5].

Not all POPs released into the environment have the same bioaccumulation pattern in different species [6]. In this study, we investigated three nondioxin-like polychlorinated biphenyls (NDL-PCBs), which do not share the dioxin's toxic mechanism, and organochlorine pesticides (OCPs) such as dichlorodiphenyltrichloroethane (DDT) and its metabolites and hexachlorocyclohexane (HCH) and its isomers. In marine mammals, POPs enter the body almost exclusively through the diet and since they are lipophilic compounds, they tend to accumulate in the lipid-rich blubber [7]. Moreover, because of the very low enzyme activities in marine mammals as compared to terrestrial animals, cetaceans have a low capacity to metabolise some persistent organic pollutants (POPs), and retain much higher concentrations of these chemicals in their fat [8, 9]. For example, out of the NDL-PCBs, congener 153 is hardly metabolised by the cytochrome P450 (CYP) enzymes, which makes this molecule extremely persistent [10]. In addition, the ability to metabolize PCB congeners is related to the levels of different families of CYP enzymes, which differ between cetaceans and other species [11]. DDT is metabolised by reductive dechlorination catalyzed by the microsomal CYP system. The principal metabolites generated are dichlorodiphenyldichloroethane (DDD) and dichlorodiphenyldichloroethylene (DDE), the latter being the most persistent and stable [12, 13]. Among HCH isomers, *β*-HCH seems to be more resistant to microbial degradation and thus more persistent in the environment than the other isomers [14]. While concentrations of OCPs (DDTs, HCB, and HCHs) in harbour porpoises stranded on the Belgian North Sea coast are low, relatively high concentrations of PCB are present [15].

Public concern about environmental contamination by POPs increased recently because of many evidences showing that some of these compounds are xenoestrogens and interact with the endocrine system, resulting in numerous biological effects that may affect the health of humans and animals [7, 16–20], as demonstrated in recent studies involving polar bears (*Ursus maritimus*) [21, 22]. It has been reported that PCBs are associated with reproductive, estrogenic and antiandrogenic, effects [23]. DDT and its metabolites bind the estrogen receptor [24], disrupting the endocrine and reproductive systems. Furthermore, HCH may also affect the reproductive system, possibly through endocrine-mediated mechanisms [25]. In the case of harbour porpoises, several studies have provided consistent support for the hypothesis of a PCB exposure induced immunosuppression contributing to infectious disease mortality [26, 27]. Furthermore, contaminants such as PCBs, DDT, and DDE may interfere with harbour porpoise thyroid functions leading to severe interfollicular fibrosis [28].

Currently, the United States Environmental Protection Agency (US-EPA) estimates that there are more than 87000 potential endocrine disrupters in the world. Nevertheless, developing methods to detect so many chemicals would take a massive financial mobilization. In this way, research on screening methods using short term bioassays to assess the risk of exposure to endocrine disrupting chemicals is needed [29, 30].

In this context, the aim of this study was to measure the hormonal activity of North Sea harbour porpoise blubber samples and to identify the compounds that contribute to the hormonal activity of these samples. In order to associate hormonal activities to xenoestrogen contamination levels, the samples were analysed both by cell-based assays (reporter gene assays) and chemical analysis (mass spectrometry coupled to gas chromatography [GC-MS]). GC-MS combines high separation power with good identification capabilities, while reporter gene assays allow the measurement of the hormonal potency of samples [31, 32], as they are based on the activation of the nuclear estrogen hormone receptor [33]. In this study, we decided to focus on the evaluation of the (anti-) estrogenic effect of xenoestrogens, as it is associated with disruption of the endocrine and reproductive systems. Studies combining biological and chemical analysis have proved to be valuable in uncovering the presence and the impact of hormone-like compounds in the wildlife [34]. Reporter gene assays were largely used to analyse samples such as harbour sediment and wasted waters [35, 36], but only few studies were conducted to assess the hormonal activity of biological tissues [37–39].

## 2. Material and Methods

### 2.1. Equipment, Chemicals, and Reagents

The following equipment and materials have been used in this study: carbon dioxide (Air Liquide, Liège, Belgium); helium (Air Products, Brussels, Belgium); acetonitrile (Biosolve, Valkenswaard, Netherlands); luminometer Orion II (BRS, Drogenbos, Belgium); adenosine triphosphate (ATP), Dulbecco's modified Eagle's medium (DMEM), DMEM without red phenol, and fetal bovine serum and trypsin (Fisher Bioblock Scientific, Tournai, Belgium); Focus gas chromatograph and Polaris Q mass spectrometer (Interscience, Louvain-la-Neuve, Belgium); charcoal, dextran,* o*,*p*′-DDD,* p*,*p*′-DDD,* p*,*p*′-DDE,* o*,*p*′-DDT,* p*,*p*′-DDT, 17*β*-estradiol, HCB, *α*-HCH, *β*-HCH, *γ*-HCH, *δ*-HCH, nonane, PCB 80 ^13^C, PCB 138 (2,2′,3,4,4′,5′-hexachlorobiphenyl), PCB 153 (2,2′,4,4′,5,5′-hexachlorobiphenyl), and PCB 180 (2,2′,3,4,4′,5,5′-heptachlorobiphenyl) (Sigma-Aldrich, Bornem, Belgium); D-luciferin (potassium salt) (Synchem OHG, Kassel, Germany); HT8 column (VWR, Leuven, Belgium).

### 2.2. Stable Reporter Cell Lines and Culture

To obtain an estrogen-responsive cell line, MCF-7 human mammary tumour cells were stably transformed with a reporter vector containing the firefly luciferase gene under control of the vitellogenin promoter [24, 40]. These cells were grown in 75 cm^2^ culture flasks in DMEM, supplemented by 10% heat-inactivated foetal bovine serum (FBS), at 37°C under 5% CO_2_.

### 2.3. Samples

Blubber samples were obtained from 12 juvenile harbour porpoises (8 males and 4 females) stranded on the Belgian and French North Sea coasts between 2000 and 2003. These animals were necropsied by the Laboratory for Oceanology of the University of Liège according to standard procedures detailed elsewhere [3]. Blubber samples were identified by an alphanumeric code and kept apart at −20°C until analysis.

### 2.4. Extraction Procedure

To separate POPs from blubber samples, we applied a solid-liquid extraction to extract fat with the POPs. This step was followed by an acid silica column chromatography to eliminate the fat. Using this method, we destroyed endogenous steroid hormones and their eventual conjugates, which were hydrolysed in presence of inorganic acids [41]. The advantage of this method is that natural hormones present in the samples could be eliminated from the extracts and would not interfere in the estrogen-like activity elicited by samples as pointed out by some authors [42].

The extraction was performed as follows. One g of harbour porpoise blubber was homogenized in a test tube containing 2 mL of hexane using a glass stirring rod. The organic phase was separated and the hexane evaporated under nitrogen until dryness. Then, 0.25 g or 0.1 g of the extracted fat was solubilised in two different tubes containing 2 mL of hexane for the estrogen receptor- (ER-) mediated activity assays and GC-MS analyses, respectively. The fat solubilised in hexane was applied to a glass column, prewashed with hexane, filled (from the bottom to top) with 5 g of acid silica (40% H_2_SO_4_ w/w), 1 g of deactivated alumina, and 1 g of Na_2_SO_4_. POPs were eluted with a mixture of dichloromethane and hexane (1 : 3), the eluent was evaporated to dryness. The residue of the first tube was recovered in 25 *μ*L of acetonitrile for the estrogen receptor- (ER-) mediated activity assays, and the residue of the second tube was recovered in 100 *μ*L of nonane containing 10 *μ*L of an internal standard (PCB 80 ^13^C 1.0 ng/*μ*L) for GC-MS analysis. In order to measure the possible loss of the estrogenic activity due to the extraction and purification procedure, a solution containing 13 POPs (see below) was submitted to the extraction/purification procedure and the extracts were then analysed by reporter gene assay. A procedure blank was performed to measure the background response in the reporter gene assay.

### 2.5. Cell-Based Assays to Test Estrogen Receptor- (ER-) Mediated Activity

The cell-based assays for estrogen receptor ER-mediated activity were carried out as follows. Ninety % confluent MCF-7-ERE cells were cultured at least 24 h in DMEM without phenol red (supplemented by 10% of FBS previously treated with charcoal-dextran), and they were released from the culture flask using 1.5 mL of trypsin (0.5 g/L). Then, cells were suspended in 10 mL of fresh culture medium and this suspension was diluted two times. One hundred *μ*L of diluted cells was seeded in 96-well culture plates, which were incubated overnight at 37°C under 5% CO_2_. Afterwards, cells were incubated with the standards or the extracts of blubber samples to be tested for 24 hours. The final volume in one well was 200 *μ*L. A 17*β*-estradiol (E2) calibration curve was performed on each plate (7.0 × 10^−5^~2.0 × 10^−1^ ng E2/mL culture medium containing 0.8% acetonitrile). To study agonistic activity of the standards or the extracts of blubber samples (ability to mimic endogenous hormones), cells were exposed, one at a time, to a selection of 13 individual POPs (*o*,*p*′-DDD,* p*,*p*′-DDD,* p*,*p*′-DDE,* o*,*p*′-DDT,* p*,*p*′-DDT, HCB, *α*-HCH, *β*-HCH, *γ*-HCH, *δ*-HCH, PCB 138, PCB 153, and PCB 180) or blubber extracts diluted in acetonitrile. Each POP was tested individually at three different concentrations: 80, 400, and 2000 ng/mL culture medium. Then, seven mixtures containing the same weight proportion of each POP (to reach final global concentrations of 80, 400, and 2000 ng/mL culture medium) were tested. The final proportion of acetonitrile in the medium in the agonistic tests was 0.4%. To study the antiestrogenic activity of the tested standards or the extracts of blubber samples (ability to inhibit the binding of a hormone to its receptor), the cells were exposed to increasing concentrations (80, 400, or 2000 ng/mL culture medium) of a selection of POPs, alone or within mixtures, or to blubber extracts diluted in acetonitrile in the presence of the reference ligand (E2) at a concentration near its EC_50_ (≈5.0 × 10^−3^ ng E2/mL culture medium). The final proportion of acetonitrile in the medium in this case was 0.8%. After incubation, the cell viability was checked under a microscope. Subsequently, the medium was removed and the cells were lysed with 50 *μ*L of lysis solution containing 25 mM of Tris, 2 mM of 1,4-dithiothreitol, 2 mM of 1,2-diaminocyclohexanetetraacetic acid, 10% of glycerol, and 1% of Triton X-100. After the addition of luciferin and ATP, the luciferase activity was determined using a luminometer and reported as relative light units (RLU). The bend points (beginning and end of the essentially linear region of the sigmoid dose-response curves) were defined as published by Sebaugh and McCray [43]. The minimal relative response was set to 17.6% to consider an accurate measurement.

### 2.6. Chemical Analyses by GC-MS

Analyses of extracts were performed using a Focus gas chromatograph coupled to a Polaris Q ion trap mass spectrometer. Helium was used as carrier gas at a flow rate of 1 mL/min. A volume of 2.0 *μ*L was injected. Separation of the target analytes was performed on a HT8 column (25 m × 0.22 mm × film thickness 0.25 *μ*m). Injector and ion source temperatures were 250°C and 220°C, respectively. The GC conditions were the following: 2 min at 120°C, ramped to 169°C (30°C/min), 13 min at 169°C, ramped to 170°C (5°C/min), 9 min at 170°C, ramped to 247°C (30°C/min), 2 min at 247°C, ramped to 320°C (20°C/min), and 2 min at 320°C. The total run time was 36 min. Spectra were acquired in single ion monitoring (SIM) mode. The* m/z* ratios scanned (with corresponding elution times) can be found in Table 1: 181, 183, 284, and 285 (11.5–16.0 min); 181 and 183 (16.0–28.0 min); 302 and 304 (28.0–30.0 min); 235, 237, 316, and 318 (30.0–31.3 min); 235, 237, 360, and 362 (31.4–33.3 min); 235, 237, 360, and 362 (31.4–33.3 min); 394 and 396 (33.3–36.0 min). For each compound, 5 calibration solutions (20, 60, 120, 240, and 400 pg/*μ*L), including the internal standard, were injected in parallel to samples extracts. The limit of detection was 1 *μ*g/kg fat.

### 2.7. Data Analysis

Cell-based assays for estrogen receptor- (ER-) mediated activity data were processed with Slide Write V6 software. Reference curves were fitted using the sigmoid dose-response curve equation: *Y* = *a*_0_/(1 + (*x*/*a*_1_)^*a*_2_^), where *x* is the concentration of E2, *Y* is the relative response ((RLU_sample_ − RLU_blank  solvent_)/(RLU_17*β*-estradiol  maximal  dose_ − RLU_blank  solvent_)), *a*_1_ is the concentration of half-maximal response (EC_50_), and *a*_2_ is the slope of the linear part of the curve. This equation was used to convert relative responses obtained for tested compounds or blubber samples extracts into estradiol equivalents (EEQ) expressed in ng/mL culture medium, for POPs standards and mixtures, and in *μ*g/kg fat, for blubber samples. Chemical analyses by GC-MS were processed using Xcalibur software (InterScience).

Student's *t*-tests were performed to determine which compounds and blubber samples produced a response significantly different from the blank in the case of agonistic tests and which compounds and blubber samples produced a response significantly different from the reference ligand (E2 at 5.0 × 10^−3^ ng/mL culture medium) in the case of antagonistic tests.

## 3. Results

### 3.1. Characterization of the MCF-7-ERE Cell Line

**Figure 1:**
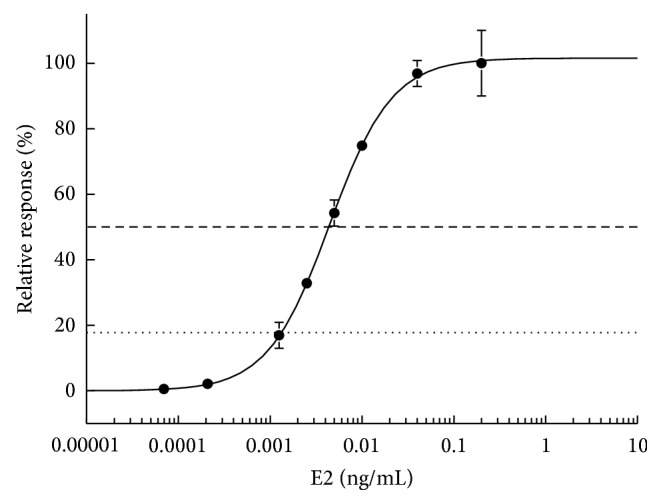
Dose-response curve of increasing concentrations of E2 (ng/mL culture medium). Data represent the mean ± S.D (*n* = 10). The maximal response observed for E2 was arbitrarily set to 100%. The dotted line represents the minimal relative response to consider an estrogenic activity (17.6% of response) and the dashed line represents EC_50_.

After exposing MCF-7-ERE cells to increasing concentrations of E2, sigmoid dose-response curves were obtained with an average coefficient of determination (*R* ^2^) of 0.99 ± 0.0049 (*n* = 10). The MCF-7-ERE cell line responded specifically to its reference ligand E2 and concentrations as low as 1.1 × 10^−3^ ng/mL of this hormone resulted in a reproducible signal, suggesting that this cell line is able to detect estrogen-like activity. The half maximal effective concentration (EC_50_) of E2 was 4.4 × 10^−3^ ± 1.5 × 10^−3^ ng/mL (Figure 1).

### 3.2. Extraction and Purification of Samples

When applying the extraction and purification procedure to a mixture of 13 POPs, we noticed that 76 ± 3% of the initial estrogenic activity was recovered. As expected, a solution containing E2 showed no estrogenic activity after being submitted to the extraction/purification procedure, indicating that E2 was undoubtedly degraded by the acidified silica of the column. The procedure blank (negative control) showed a relative response of 7%, below the threshold of 17.6%, needed to evidence an estrogenic activity. This indicates that the extraction/purification method does not induce any estrogenic activity and is thus compatible with the reporter gene assay. Likewise, when E2 was added to the extract obtained from the procedure blank, MCF-7-ERE cells responded positively and in the expected intensity, confirming that the extraction and purification methods do not bring any inhibiting effect to the cells (data not shown).

### 3.3. Detection of Estrogen Receptor- (ER-) Mediated Activity of POPs in MCF-7-ERE Cells

For each selected compound, the maximum relative response was obtained at the concentration of 2000 ng/mL culture medium (Table 2), with no apparent cytotoxicity when observing the cells under the microscope. None of the tested PCBs was able to induce a cell response above 17.6%. The five compounds belonging to the group of DDT and its metabolites presented a strong estrogenic activity, especially* o*,*p*′-DDT and* o*,*p*′-DDD, which produced a maximum relative response higher than 60%. Among the hexachloro compounds, *β*-HCH and *δ*-HCH were the compounds with the strongest estrogenic activity, with responses of 64 and 20%, respectively, at the concentration of 2000 ng/mL culture medium (Table 2).

In order to detect a possible interaction between the chemicals mentioned before, MCF-7-ERE cells were also exposed to mixtures containing the same weight proportion of each molecule included in the mixture to reach a final concentration of 80 ng/mL, 400 ng/mL, or 2000 ng/mL culture medium for the sum of all compounds. The mixture of the 3 PCBs (mixture #1) did not activate the estrogen receptor. The most concentrated mixture of DDT and its metabolites (mixture #2, 2000 ng/mL) contains each congener at a concentration of 400 ng/mL, at which only* o*,*p*′-DDT and* o*,*p*′-DDD showed an activity (relative responses of 67% and 29% for* o*,*p*′-DDT and* o*,*p*′-DDD, resp.). However, the response observed for this mixture remains below the response of* o*,*p*′-DDT alone. Similar observation can be made for the mixture of HCH isomers and HCB (mixture #3), the response of the mixture (containing each compound at a concentration of 400 ng/mL) being lower (23%) than the response of the only compound (*β*-HCH) giving a response (35%) at the concentration of 400 ng/mL. When mixing the mixtures (binary or ternary mixtures, see the four last lines of Table 2), the response obtained corresponds to the response of the most potent mixture included in the “mixture of mixtures.”

None of the selected chemicals showed antiestrogenic activity. Conversely,* o*,*p*′-DDT,* o*,*p*′-DDD, *β*-HCH, *δ*-HCH, and HCB were able to increase the cellular response when exposed simultaneously to E2 (*p* < 0.05). In this case the predicted theoretical responses for the exposure of MCF-7-ERE cells to E2 (5.0 × 10^−3^ ng/mL culture medium) simultaneously with* o*,*p*′-DDT,* o*,*p*′-DDD, or *β*-HCH (2000 ng/mL culture medium) would reach the plateau of the cell response. However, the measured responses were below 100% (data not shown).

### 3.4. Organochlorine Pollutants Determination in North Sea Harbour Porpoise Blubber by Chemical Analyses

**Figure 2:**
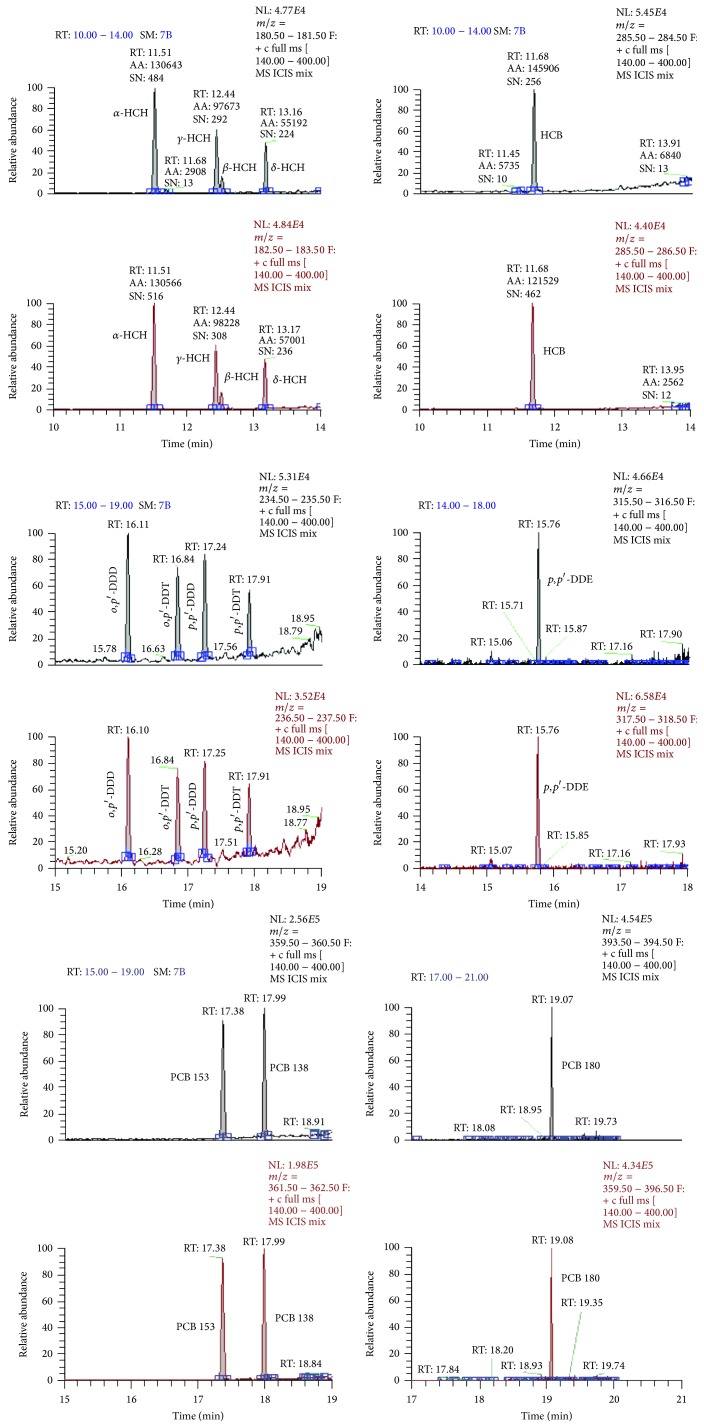
Chromatogram of a mixture of 13 standard solutions of target pollutants.

Three NDL-PCB congeners (PCB 138, PCB 153, and PCB 180), four HCH isomers (*α*-HCH, *β*-HCH, *γ*-HCH, and *δ*-HCH), HCB, and five components of the DDT group (*o*,*p*′-DDD,* p*,*p*′-DDD,* p*,*p*′*-*DDE,* o*,*p*′-DDT, and* p*,*p*′-DDT) were quantified in North Sea harbour porpoise blubber samples. After optimisation of GC-MS parameters, the obtained chromatograms presented separated and resolved peaks (Figure 2), which permitted the identification and quantification of the target compounds.

Among the chemicals analysed, the PCBs were the main contaminants in North Sea harbour porpoise blubber, followed by the DDT group and finally by the isomers of HCH and HCB (Table 3). Within each group, PCB 153 (6.0 × 10^2^ to 4.2 × 10^4^ *μ*g/kg fat),* p*,*p*′-DDE (5.1 × 10^2^ to 8.6 × 10^3^ *μ*g/kg fat), *γ*-HCH (8.0 × 10^1^ to 4.8 × 10^2^ *μ*g/kg fat), and HCB (7.6 × 10^1^ to 1.5 × 10^3^ *μ*g/kg fat) were the compounds found in largest quantities.

### 3.5. Detection of Estrogen Receptor- (ER-) Mediated Activity of North Sea Harbour Porpoise Blubber Samples

**Figure 3:**
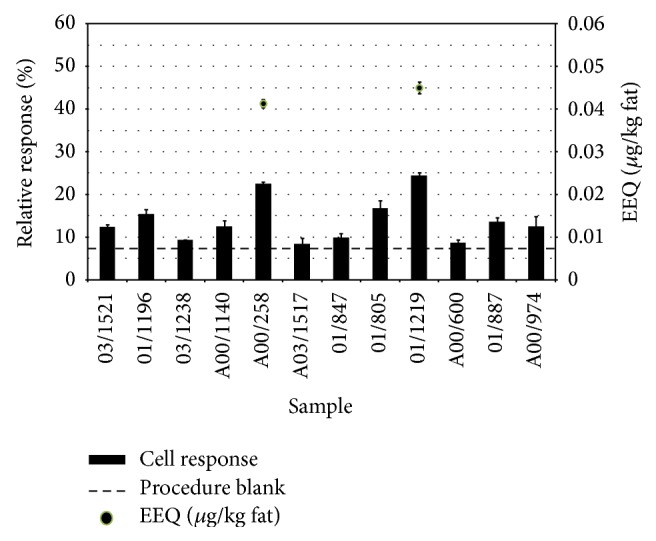
Estrogen receptor- (ER-) mediated agonistic activity elicited by 12 North Sea harbour porpoise blubber samples in MCF-7-ERE cells. The horizontal dashed line represents the response of the procedure blank. Results are expressed as percent of the maximal response induced by E2 (columns) or as *μ*g EEQ/kg fat (dots), only for the two samples showing a response significantly different from the procedure blank (*p* < 0.05).

**Figure 4:**
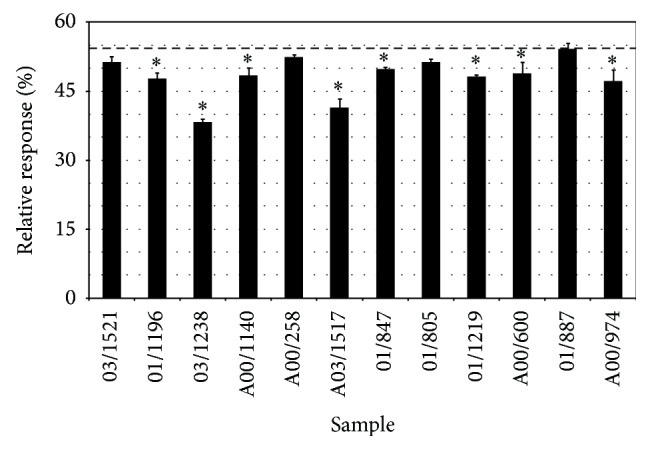
Estrogen receptor- (ER-) mediated antagonistic activity elicited by North Sea harbour porpoise blubber samples (*n* = 12) in MCF-7-ERE cells when exposed simultaneously to E2 (5.0 × 10^−3^ ng/mL). The horizontal dashed line represents the response of the cells exposed to 5.0 × 10^−3^ ng/mL of E2. Results are expressed as percent of the maximal response induced by E2. Asterisks indicate that the response is significantly different from the response of 5.0 × 10^−3^ ng/mL of E2 (*p* < 0.05).

The agonistic and antagonistic activity mediated by the estrogen receptor (ER) elicited by North Sea harbour porpoise blubber samples in MCF-7-ERE cells is reported in Figures 3 and 4, respectively. Two samples showed significant agonistic ER-mediated responses: A00/258 and 01/1219 (response significantly different from procedure blank, with *p* < 0.05). The highest response measured was 24% of the activity induced by the reference E2 (sample 01/1219), corresponding to 0.045 *μ*g EEQ/kg fat (Figure 3). When exposed simultaneously to E2, eight samples inhibited significantly (*p* < 0.05) the activity of the E2 reference ligand: 01/1169, 03/1238, A00/1140, A03/1517, 01/847, 01/1219, A00/600, and A00/974. The highest antiestrogenic effect was observed for samples 03/1238, which decreased the cellular response of 16% (Figure 4).

### 3.6. Correlation between Xenoestrogens in North Sea Harbour Porpoise Blubber and Estrogen Receptor- (ER-) Mediated Activity

From GC-MS data of sample contamination (Table 3), the amount of organochlorine contained in culture medium at the moment of the cell-based assay of blubber samples was calculated (Table 4). When comparing this result to the estrogenic activity of the individual compounds and their mixtures (Table 2), it appeared that none of the samples contained enough of the 13 measured organochlorines to elicit a positive response in the reporter gene assay, either an agonistic or antagonistic, making it not possible to predict the ER-mediated activity of the samples. Indeed, if we look to the levels of HCH isomers and HCB, they are all below 400 ng/mL culture medium, which is the smallest concentration tested for individual compounds and at which no response was recorded for these organochlorines in the cell-based assay. From the group of DDT, the most potent substances (*o*,*p*′-DDT and* o*,*p*′-DDD) represented only a minor part of the organochlorine contamination, resulting in levels below 80 ng/mL culture medium in the cell-based assay. However, one sample showed estrogenic activity, seven samples showed antiestrogenic activity, and one sample showed both estrogenic and antiestrogenic activity.

As previously shown, pollutants from the DDT group presented higher estrogenic activities than the other POPs assessed. Moreover, the most potent agonist blubber samples (A00/258 and 01/1219) presented lower Σ_PCB_/Σ_DDT_ ratios than the sample that inhibited the activity of the natural hormone the most (03/1238). This fact may explain the hormonal activity of samples A00/258 and 01/1219.

The most contaminated sample (A03/1517), containing a total of more than 88 mg organochlorine/kg fat, displayed a slight antiestrogenic effect. This can be easily explained by the high contribution of PCBs (more than 81 mg/kg), which were not inducing any response in the estrogen-responsive cells.

## 4. Discussion

The study of estrogen receptor- (ER-) mediated activity of POPs in MCF-7-ERE cells showed that several organochlorine pollutants (DDT and metabolites and HCH isomers) present an estrogenic activity, which was also confirmed by other authors [24, 44–47]. The fact that luciferase gene expression was not induced by PCB 153 and PCB 180 confirms the study of Plíšková et al. [48], where it was observed that higher-chlorinated PCB congeners present low estrogenic activity. It seems that the activity of ER agonism of a PCB is linked to its structure, as larger estrogenic potencies have been reported for low chlorinated compounds [49]. These assays also point out how complex the prediction of the effect of a POP mixture can be. In fact, the approach to investigate endocrine disrupters within mixtures is a new tool that provides clear evidence that POPs have different effects when they are not alone, suggesting that risk assessment should take into consideration the effect of these chemicals within mixture rather than their individual effects [50–54].

Concentrations of organochlorine chemicals found in North Sea harbour porpoise blubber samples are comparable with data formerly published [5, 6, 55, 56]. Percentage distribution of PCB congeners was constant between samples and the importance of single congeners was as follows: 153 > 138 > 180. Similar profiles were found in various harbour porpoise tissues in previous studies [10, 15, 57, 58]. The distribution pattern of PCBs can be explained by differences in structural characteristics within PCB congeners that determine whether a molecule can be easily metabolised by cytochrome P450 enzymes or not.

Metabolites play a dominant role if their persistence exceeds that of the parent product. Even though DDT was banned from utilisation in North America and Western Europe in the 1970s with no new input in the southern North Sea during the last decades [59], the breakdown products of DDT were still detected in the analyzed samples (DDE showing the highest concentrations). In fact, DDT is rapidly metabolised to DDD and slowly metabolised to DDE. Furthermore, DDD has a significantly higher elimination rate than DDE and, subsequently, aquatic animals retain more DDE in their bodies [60, 61]. Thus, the detected contents of DDD and DDE determined in our samples are possibly due to the breakdown of DDT in the environment.

The high concentration of *γ*-HCH in samples is surprising as *γ*-HCH is relatively rapidly phototransformed to *α*-HCH in the environment [62]. This same profile was found in other species of the North Sea [63]. The high concentration of *γ*-HCH found in blubber samples may indicate a current utilization of this pesticide in some countries, with a subsequent global scale pollution since both the atmosphere and the ocean are important transport media for HCH isomers [64, 65]. Also, HCH isomers and HCB were detected in the samples in lower concentration than PCB congeners and pollutants from the DDT group, possibly because these compounds are easily metabolized and are more water soluble [66].

The large difference of contamination level between samples is probably due to a possible difference in health conditions (e.g., disease or parasitic infection) of the animals, as reported by Pierce et al. [7], maternal transfer, age, and diet. As shown in Table 3, samples from animals that did not present emaciation or parasitic infection figured among the least contaminated. In addition, it was reported that POP concentration in juvenile marine mammals is much higher than in the mothers, since a large portion of the mother's POP burden, transferred to the calf during gestation and lactation, is assimilated into its blubber and other tissues [67], placing juveniles at higher risk [55].

The analyses of blubber samples by cell-based bioassays suggest that POPs measured by GC-MS in this study cannot justify alone the estrogenicity of the extracts and that other endocrine disrupters contaminate the porpoises. Interestingly, the sample showing the highest estrogenic activity and the two samples displaying the highest ER antagonistic activities are among the most contaminated samples. It can be expected that these samples, with a high load of contaminants, also contain other POPs than the 13 organochlorine measured in this study, among which some are agonist or antagonist of the estrogen receptor, such as dioxins and furans [68].

## 5. Conclusions

Our study permitted the analysis of harbour porpoise blubber samples both by chemical analysis and cell-based assays, providing consistent data including the level of contamination and the (anti-) estrogenic activity of these samples. Within each group of studied substances, PCB 153,* p*,*p*′-DDE, and HCB were the compounds found in highest concentrations. Two samples showed estrogenic activity, seven samples showed antiestrogenic activity, and one sample showed both estrogenic and antiestrogenic activity. However, our results suggest that the 13 POPs measured by GC-MS in the samples cannot explain alone the estrogenicity of the extracts and that other EDCs contaminate the porpoises.

High load persistent organic pollutants in porpoise blubber samples indicate that these pollutants can also be found in food from the North Sea, and the hormonal activity measured in some samples confirms the presence of endocrine disrupting chemicals in the marine environment.

## Figures and Tables

**Table 1:** *m*/*z* ratios scanned and corresponding windows of elution times for each target compound.

*m*/*z* ratios	Elution time (min)	Target compound
181/183	11.5–16.0	*α*-HCH
284/285	11.5–16.0	HCB
181/183	16.0–28.0	*γ*-HCH, *β*-HCH, and *δ*-HCH
302/304	28.0–30.0	PCB 80 ^13^C
235/237	30.0–31.4	*o*,*p*′-DDD
316/318	30.0–31.4	*p*,*p*′-DDE
235/237	31.4–33.3	*o*,*p*′-DDT, *p*,*p*′-DDD, and *p*,*p*′-DDT
360/362	31.4–33.3	PCB 153 and PCB 138
394/396	33.3–36.0	PCB 180

**Table 2:** Estrogen receptor-mediated luciferase expression (measured as light emission) observed in MCF-7-ERE cells exposed during 24 hours to single compounds or mixtures of selected POPs. All mixtures contained the same weight proportion of the constituents to achieve the concentration of 80 ng/mL, 400 ng/mL, or 2000 ng/mL medium (*n* = 3).

	Relative response (%)^a^			EEQ (ng/mL)^b^		
	80 ng/mL	400 ng/mL	2000 ng/mL	80 ng/mL	400 ng/mL	2000 ng/mL
PCB 138	<LOQ	<LOQ	<LOQ	n/a	n/a	n/a
PCB 153	<LOD	<LOD	<LOQ	n/a	n/a	n/a
PCB 180	<LOD	<LOD	<LOD	n/a	n/a	n/a
Mixture of PCB congeners (1)	<LOD	<LOD	<LOQ	n/a	n/a	n/a

*o*,*p*′-DDT	28 ± 0	67 ± 3	72 ± 7	2.1 ± 0.0 × 10^−3^	7.6 ± 0.8 × 10^−3^	9.4 ± 2.4 × 10^−3^
*o*,*p*′-DDD	<LOD	29 ± 3	66 ± 3	n/a	2.1 ± 0.2 × 10^−3^	7.3 ± 0.6 × 10^−3^
*p*,*p*′-DDT	<LOD	<LOQ	36 ± 2	n/a	n/a	2.8 ± 0.2 × 10^−3^
*p*,*p*′-DDD	<LOD	<LOQ	32 ± 2	n/a	n/a	2.4 ± 0.2 × 10^−3^
*p*,*p*′-DDE	<LOD	<LOQ	21 ± 1	n/a	n/a	1.5 ± 0.0 × 10^−3^
Mixture of DDT and its metabolites (2)	<LOQ	26 ± 1	49 ± 4	n/a	1.9 ± 0.1 × 10^−3^	4.3 ± 0.5 × 10^−3^

*β*-HCH	<LOQ	35 ± 2	64 ± 4	n/a	2.7 ± 0.1 × 10^−3^	6.8 ± 1.0 × 10^−3^
*δ*-HCH	<LOQ	<LOQ	20 ± 3	n/a	n/a	1.4 ± 0.2 × 10^−3^
*γ*-HCH	<LOD	<LOQ	<LOQ	n/a	n/a	n/a
*α*-HCH	<LOD	<LOQ	<LOQ	n/a	n/a	n/a
HCB	<LOD	<LOD	<LOD	n/a	n/a	n/a
Mixture of HCH isomers and HCB (3)	<LOQ	<LOQ	23 ± 1	n/a	n/a	1.7 ± 0.1 × 10^−3^

Mixture (1) + (2)	<LOQ	32 ± 1	52 ± 2	n/a	2.4 ± 0.1 × 10^−3^	4.7 ± 0.3 × 10^−3^
Mixture (1) + (3)	<LOQ	<LOQ	28 ± 2	n/a	n/a	2.1 ± 0.2 × 10^−3^
Mixture (2) + (3)	<LOQ	20 ± 7	54 ± 2	n/a	1.5 ± 0.5 × 10^−3^	5.0 ± 0.3 × 10^−3^
Mixture (1) + (2) + (3)	<LOQ	27 ± 1	54 ± 2	n/a	2.0 ± 0.1 × 10^−3^	5.0 ± 0.3 × 10^−3^

^a^The maximal response observed for E2 was arbitrarily set to 100% and the responses observed for the chemicals and mixtures are expressed in percentage of the maximal response (relative response).

^b^Estradiol equivalents were determined by linear extrapolation from calibration curves obtained after exposure to E2 and are expressed in ng/mL culture medium.

n/a: not applicable.

**Table 3:** Gender, length (cm), weight (kg), blubber thickness (cm), and blubber levels (*μ*g/kg) of 13 organochlorine compounds of 12 North Sea harbour porpoise individuals. Chemicals were divided in three groups and the sum of concentrations for each group is presented for the different samples.

	03/1521	01/1196	03/1238	A00/1140	A00/258	A03/1517	01/847	01/805	01/1219	A00/600	01/887	A00/974	Average
Gender	M	M	F	F	M	M	F	M	M	M	M	F	—
Length	117	104	127	112	108	128	114	99	112	103	110	114	112 ± 9
Weight	30	n/a	26	20	18	22	29	14	19	27	17	22	22 ± 5
Blubber thickness	20	22	10	13	10	8	20	5	8	40	6	20	15 ± 10
Emaciation	n/a	n/a	Yes	Yes	Yes	Yes	No	Yes	Yes	No	Yes	No	—
Parasites	n/a	n/a	Yes	Yes	Yes	Yes	No	No	Yes	No	Yes	No	—

PCB 138	9.9 × 10^2^	1.3 × 10^3^	8.3 × 10^3^	3.8 × 10^3^	3.7 × 10^3^	3.1 × 10^4^	2.7 × 10^3^	3.7 × 10^3^	7.7 × 10^3^	3.6 × 10^3^	2.3 × 10^3^	5.9 × 10^2^	5.8 ± 8.3 × 10^3^
PCB 153	1.4 × 10^3^	1.7 × 10^3^	1.2 × 10^4^	6.3 × 10^3^	5.5 × 10^3^	4.2 × 10^4^	4.0 × 10^3^	5.5 × 10^3^	1.1 × 10^4^	5.1 × 10^3^	3.2 × 10^3^	6.0 × 10^2^	0.8 ± 1.1 × 10^4^
PCB 180	3.3 × 10^2^	3.2 × 10^2^	2.5 × 10^3^	1.8 × 10^3^	1.2 × 10^3^	7.4 × 10^3^	8.0 × 10^2^	1.2 × 10^3^	1.9 × 10^3^	9.3 × 10^2^	7.9 × 10^2^	1.1 × 10^2^	1.6 ± 1.9 × 10^3^
Σ (3) PCBs	2.7 × 10^3^	3.3 × 10^3^	2.3 × 10^4^	1.2 × 10^4^	1.0 × 10^4^	8.1 × 10^4^	7.5 × 10^3^	1.0 × 10^4^	2.1 × 10^4^	9.6 × 10^3^	6.3 × 10^3^	1.3 × 10^3^	1.6 ± 2.2 × 10^4^

*o*,*p*′-DDT	4.8 × 10^1^	1.0 × 10^2^	3.4 × 10^2^	9.8 × 10^1^	7.4 × 10^1^	2.1 × 10^2^	6.0 × 10^1^	8.2 × 10^1^	8.6 × 10^2^	5.4 × 10^1^	1.7 × 10^2^	7.1 × 10^1^	1.8 ± 2.3 × 10^2^
*o*,*p*′-DDD	n/d	7.2 × 10^1^	1.8 × 10^2^	7.8 × 10^1^	6.8 × 10^1^	4.1 × 10^2^	n/d	8.6 × 10^1^	5.2 × 10^2^	n/d	6.9 × 10^1^	4.0 × 10^1^	1.7 ± 1.8 × 10^2^
*p*,*p*′-DDT	1.8 × 10^2^	3.2 × 10^2^	7.9 × 10^2^	5.6 × 10^2^	4.7 × 10^2^	7.1 × 10^2^	1.9 × 10^2^	3.7 × 10^2^	1.4 × 10^3^	2.0 × 10^2^	5.8 × 10^2^	1.1 × 10^2^	4.9 ± 3.6 × 10^2^
*p*,*p*′-DDD	3.4 × 10^2^	5.3 × 10^2^	8.9 × 10^2^	9.2 × 10^2^	8.5 × 10^2^	1.2 × 10^3^	2.4 × 10^2^	7.3 × 10^2^	2.6 × 10^3^	4.1 × 10^2^	6.0 × 10^2^	1.4 × 10^2^	7.9 ± 6.6 × 10^2^
*p*,*p*′-DDE	1.0 × 10^3^	1.2 × 10^3^	3.1 × 10^3^	2.6 × 10^3^	2.9 × 10^3^	4.1 × 10^3^	9.1 × 10^2^	2.4 × 10^3^	8.6 × 10^3^	1.4 × 10^3^	2.3 × 10^3^	5.1 × 10^2^	2.6 ± 2.2 × 10^3^
Σ (5) DDTs	1.6 × 10^3^	2.3 × 10^3^	5.3 × 10^3^	4.3 × 10^3^	4.4 × 10^3^	6.6 × 10^3^	1.4 × 10^3^	3.7 × 10^3^	1.4 × 10^4^	2.1 × 10^3^	3.7 × 10^3^	8.6 × 10^2^	4.2 ± 3.5 × 10^3^

*β*-HCH	n/d	n/d	9.7 × 10^1^	n/d	8.8 × 10^1^	8.0 × 10^1^	n/d	8.3 × 10^1^	1.3 × 10^2^	4.2 × 10^1^	7.4 × 10^1^	4.7 × 10^1^	8.0 ± 2.7 × 10^1^
*δ*-HCH	n/d	n/d	n/d	n/d	n/d	n/d	n/d	n/d	n/d	n/d	n/d	n/d	—
*γ*-HCH	9.8 × 10^1^	1.4 × 10^2^	1.3 × 10^2^	1.3 × 10^2^	2.4 × 10^2^	2.0 × 10^2^	8.0 × 10^1^	1.7 × 10^2^	4.8 × 10^2^	1.7 × 10^2^	1.5 × 10^2^	1.2 × 10^2^	1.8 ± 1.1 × 10^2^
*α*-HCH	7.0 × 10^1^	n/d	6.8 × 10^1^	6.6 × 10^1^	n/d	n/d	n/d	8.0 × 10^1^	9.8 × 10^1^	7.1 × 10^1^	1.0 × 10^2^	7.4 × 10^1^	7.9 ± 1.4 × 10^1^
HCB	1.4 × 10^2^	2.1 × 10^2^	5.4 × 10^2^	2.2 × 10^2^	3.1 × 10^2^	4.9 × 10^2^	1.3 × 10^2^	3.4 × 10^2^	1.5 × 10^3^	9.8 × 10^1^	6.0 × 10^2^	7.6 × 10^1^	3.9 ± 4.0 × 10^2^
Σ (5) HCHs + HCB	3.1 × 10^2^	3.5 × 10^2^	8.4 × 10^2^	4.2 × 10^2^	6.3 × 10^2^	7.7 × 10^2^	2.1 × 10^2^	6.7 × 10^2^	2.2 × 10^3^	3.9 × 10^2^	9.3 × 10^2^	3.1 × 10^2^	6.7 ± 5.4 × 10^2^

Σ (13) total	4.6 × 10^3^	5.9 × 10^3^	2.9 × 10^4^	1.7 × 10^4^	1.5 × 10^4^	8.8 × 10^4^	9.1 × 10^3^	1.5 × 10^4^	3.7 × 10^4^	1.2 × 10^4^	1.1 × 10^4^	2.5 × 10^3^	2.1 ± 2.4 × 10^4^

n/d: not detected; n/a: not available.

**Table 4:** Concentrations (ng/mL) of 13 organochlorine compounds in the extracts of 12 samples of North Sea harbour porpoise blubber, in culture medium solution to which the ER sensitive cells were exposed in the cell based assay (calculated from GC-MS data from Table 3; according to that 1 mL of culture medium contains the contaminants extracted from 0.04 g of fat). For the samples displaying an estrogenic activity, the percentage of relative response compared to the maximal response induced by E2 is indicated between brackets.

	03/1521	01/1196	03/1238	A00/1140	A00/258	A03/1517	01/847	01/805	01/1219	A00/600	01/887	A00/974
PCB 138	4.0 × 10^1^	5.2 × 10^1^	3.3 × 10^2^	1.5 × 10^1^	1.5 × 10^2^	1.3 × 10^3^	1.1 × 10^2^	1.5 × 10^2^	3.1 × 10^2^	1.4 × 10^2^	9.1 × 10^1^	2.4 × 10^1^
PCB 153	5.4 × 10^1^	6.7 × 10^1^	4.9 × 10^2^	2.5 × 10^2^	2.2 × 10^2^	1.7 × 10^3^	1.6 × 10^2^	2.2 × 10^2^	4.5 × 10^2^	2.1 × 10^2^	1.3 × 10^2^	2.4 × 10^1^
PCB 180	1.3 × 10^1^	1.3 × 10^1^	1.0 × 10^2^	7.3 × 10^1^	4.7 × 10^1^	3.0 × 10^2^	3.2 × 10^1^	4.9 × 10^1^	7.5 × 10^1^	3.7 × 10^1^	3.1 × 10^1^	4.5 × 10^0^
Σ (3) PCBs	1.1 × 10^2^	1.3 × 10^2^	9.2 × 10^2^	4.8 × 10^2^	4.2 × 10^2^	3.2 × 10^3^	3.0 × 10^2^	4.2 × 10^2^	8.4 × 10^2^	3.9 × 10^2^	2.5 × 10^2^	5.2 × 10^1^

*o*,*p*′-DDT	1.9 × 10^0^	4.2 × 10^0^	1.4 × 10^1^	3.9 × 10^0^	3.0 × 10^0^	8.5 × 10^0^	2.4 × 10^0^	3.3 × 10^0^	3.4 × 10^1^	2.2 × 10^0^	6.7 × 10^0^	2.8 × 10^0^
*o*,*p*′-DDD	n/a	2.9 × 10^0^	7.2 × 10^0^	3.1 × 10^0^	2.7 × 10^0^	1.7 × 10^1^	n/a	3.4 × 10^0^	2.1 × 10^1^	n/a	2.8 × 10^0^	1.6 × 10^0^
*p*,*p*′-DDT	7.2 × 10^0^	1.3 × 10^1^	3.2 × 10^1^	2.2 × 10^1^	1.9 × 10^1^	2.8 × 10^1^	7.5 × 10^0^	1.5 × 10^1^	5.6 × 10^1^	8.0 × 10^0^	2.3 × 10^1^	4.3 × 10^0^
*p*,*p*′-DDD	1.4 × 10^1^	2.1 × 10^1^	3.5 × 10^1^	3.7 × 10^1^	3.4 × 10^1^	5.0 × 10^1^	9.5 × 10^0^	2.9 × 10^1^	1.0 × 10^2^	1.6 × 10^1^	2.4 × 10^1^	5.6 × 10^0^
*p*,*p*′-DDE	4.2 × 10^1^	5.0 × 10^1^	1.2 × 10^2^	1.1 × 10^2^	1.2 × 10^2^	1.6 × 10^2^	3.6 × 10^1^	9.6 × 10^1^	3.4 × 10^2^	5.6 × 10^1^	9.3 × 10^1^	2.0 × 10^1^
Σ (5) DDTs	6.4 × 10^1^	9.1 × 10^1^	2.1 × 10^2^	1.7 × 10^2^	1.8 × 10^2^	2.7 × 10^2^	5.6 × 10^1^	1.5 × 10^2^	5.6 × 10^2^	8.2 × 10^1^	1.5 × 10^2^	3.5 × 10^1^

*β*-HCH	n/a	n/a	3.9 × 10^0^	n/a	3.5 × 10^0^	3.2 × 10^0^	n/a	3.3 × 10^0^	5.0 × 10^0^	1.7 × 10^0^	3.0 × 10^0^	1.9 × 10^0^
*δ*-HCH	n/a	n/a	n/a	n/a	n/a	n/a	n/a	n/a	n/a	n/a	n/a	n/a
*γ*-HCH	3.9 × 10^0^	5.8 × 10^0^	5.3 × 10^0^	5.1 × 10^0^	9.4 × 10^0^	7.8 × 10^0^	3.2 × 10^0^	6.9 × 10^0^	1.9 × 10^1^	7.0 × 10^0^	6.1 × 10^0^	4.6 × 10^0^
*α*-HCH	2.8 × 10^0^	n/a	2.7 × 10^0^	2.6 × 10^0^	n/a	n/a	n/a	3.2 × 10^0^	3.9 × 10^0^	2.8 × 10^0^	4.2 × 10^0^	3.0 × 10^0^
HCB	5.7 × 10^0^	8.4 × 10^0^	2.1 × 10^1^	8.9 × 10^0^	1.2 × 10^1^	2.0 × 10^1^	5.4 × 10^0^	1.3 × 10^1^	6.1 × 10^1^	3.9 × 10^0^	2.4 × 10^1^	3.0 × 10^0^
Σ (5) HCHs + HCB	1.2 × 10^1^	1.4 × 10^1^	3.3 × 10^1^	1.7 × 10^1^	2.5 × 10^1^	3.1 × 10^1^	8.6 × 10^0^	2.7 × 10^1^	8.9 × 10^1^	1.5 × 10^1^	3.7 × 10^1^	1.3 × 10^1^

Σ (13) total	1.9 × 10^2^	2.4 × 10^2^	1.2 × 10^3^	6.7 × 10^2^	6.2 × 10^2^	3.5 × 10^3^	3.7 × 10^2^	5.9 × 10^2^	1.5 × 10^3^	4.8 × 10^2^	4.4 × 10^2^	9.9 × 10^1^

Activity recorded in the ER reporter gene assay (% of relative response)	None	Antiestrogenic (7%)	Antiestrogenic (16%)	Antiestrogenic (6%)	Estrogenic (22%)	Antiestrogenic (13%)	Antiestrogenic (5%)	None	Estrogenic (24%) and antiestrogenic (6%)	Antiestrogenic (5%)	None	Antiestrogenic (7%)

n/a: not applicable.
